# An overview of the sexual and reproductive health status and service delivery among Syrian refugees in Jordan, nine years since the crisis: a systematic literature review

**DOI:** 10.1186/s12978-020-01005-7

**Published:** 2020-10-28

**Authors:** Mirwais Amiri, Ieman M. El-Mowafi, Tala Chahien, Hind Yousef, Loulou Hassan Kobeissi

**Affiliations:** 1 The Eastern Mediterranean Public Health Network (EMPHNET), Shmeisani, Abdallah Ben Abbas Street, Bldg No. 42, Amman, Jordan; 2 Faculty of Health Sciences, The University of Ottawa, 75 Laurier Ave E, Ottawa, ON K1N 6N5 Canada; 3 World Health Organization, 20 Avenue Appia, 1211, Geneva 27, Switzerland

**Keywords:** Sexual and reproductive health, Reproductive health, Sexual violence, Family violence, Intimate partner violence, Gender-based violence, Minimum initial service package, Syrian refugees, Syrian crisis, Humanitarian settings

## Abstract

**Background:**

The Syrian refugee crisis has led to massive displacement into neighboring countries including Jordan. This crisis has caused a significant strain on the sexual and reproductive health (SRH) services to the host communities and Syrian refugees. The Minimum Initial Service Package (MISP) is a standard package of services that should be implemented at the onset of an emergency. Due to their importance in protracted humanitarian crisis, this systematic review aimed to assess the utilization of SRH and MISP after 9 years of the crisis.

**Methods:**

We searched PubMed, Medline/Ovid and Scopus for both quantitative and qualitative studies from 1 January 2011 to 30 November 2019. Our search included both free text key words and Medical Subject Headings (MeSH) for various forms and acronmym of the following terms: (Sexual and) Reproductive Health, Sexual/Gender-based/Family/Intimate partner violence, Minimum Initial Service Package, MISP, Women, Girls, Adolescents, Syrian, Refugee, Jordan, Humanitarian crisis, War, (armed) conflict, and Disaster. Boolean operators and star truncation (*) were used as needed. We further conducted an in-depth review of the available grey literature published during the same timeframe. Using a narrative synthesis approach, two authors independently extracted and analyzed data from published papers. After removal of duplicates, screening, and assessing for eligibility of 161 initially identified citations, 19 papers were selected for review.

**Results:**

Findings from this review indicated a number of barriers to access, utilization, and implementation of SRH services, including lack of reliable information on sexual and gender-based violence (SGBV), aggravation of early marriages by crisis setting, gaps in the knowledge and use of family planning services, inadequate STIs and HIV coverage, and some issues around the provision of maternal health services.

**Conclusion:**

The findings from this review are suggestive of a number of barriers pertaining to access, utilization, and implementation of SRH services. This is especially true for transitioning from MISP to comprehensive SRH services, and particularly for refugees outside camps. Following are needed to address identified barriers: improved inter-agency coordination, better inclusion/engagement of local initiatives and civil societies in SRH services delivery, improved quality of SRH services, adequate and regular training of healthcare providers, and increased awareness of Syrian women and adolescent girls. Also, more implementing research is required to identify ways to transition SRH provision from the MISP to comprehensive care for the Syrian refugee population in Jordan.

## Plain English summary

In order to understand better the sexual and reproductive health (SRH) needs of Syrian refugees, our study team aimed to review all published papers and available reports in an attempt to reduce research waste.

The purpose of this review is to examine the available published literature on the provision of SRH services for Syrian refugees in Jordan. We specifically aimed to identify and describe the current SRH issues and challenges faced by Syrian refugees in Jordan. We sought to understand better about service delivery in Jordan for Syrian refugees, its ability to transition to comprehensive SRH services, and highlight prevailing bottlenecks and opportunities impacting this transition. Therefore, we aimed to conduct a scoping review to systematically search databases for published articles.

Our findings indicate that Syrian refugee women face multi-faceted barriers to access and uptake of SRH services. These barriers include lack of reliable information on sexual and gender-based violence (SGBV), aggravation of early marriages by crisis setting, gaps in the knowledge and use of family planning services, inadequate STI and HIV coverage, and issues around the provision of maternal health services. The following are needed to address identified barriers: improved inter-agency coordination, better inclusion/engagement of local initiatives and civil societies in SRH services delivery, improved quality of SRH services, adequate and regular training of healthcare providers, and increased awareness of Syrian women and adolescent girls.

## Background

The Syrian crisis has resulted in one of the worst humanitarian disasters in modern history. Since 2011, the crisis had led to over 13 million people requiring humanitarian assistance; more than 11 million Syrians are currently internally displaced or have sought refuge into neighboring countries; half of whom are women and girls of reproductive age. The United Nations High Commission for Refugees (UNHCR) estimates that over one million people from Syria currently reside in Jordan, including approximately 650,000 Syrian refugees registered with UNHCR [1, 2], and as many as 700,000 unregistered individuals who are unable to return to Syria [2]. The overwhelming majority of Syrian refugees in Jordan live in rural and urban areas across the country; indeed, only 21% of Syrian refugees live in camp-based settings. As a result, Syrians who live in these disparate communities must rely on local health services [3]. Evidence suggests that the influx of refugees has placed an unsustainable strain on schools, housing, sanitation, food and the health care systems in Jordan [4]. Further, it is estimated that 86% of Syrian refugees are currently living under the Jordanian poverty line and 75% are considered highly shelter vulnerable [5]. The inequitable distribution of poverty, employment opportunities, and educational attainment, which are further exacerbated by displacement, impacts individuals and communities’ access to health services and health outcomes [6].

A body of evidence shows that Syrian refugee women and girls have significant unmet sexual and reproductive health (SRH) needs in comparison to their host community counterparts [7]. According to the 2018 Jordan’s Population and Family Health Survey (JPFHS), Syrian refugee women have a total fertility rate (TFR) of 4.7 versus 2.6 children per women among Jordanian women [8]. Further, several recent studies suggest that Syrian refugee women and girls experience high rates of sexual violence and early and forced marriage, have more difficulty accessing family planning services and in turn experience higher rates of unintended and unwanted pregnancy [7, 9, 10]. Consistent with global trends, displacement has resulted in many families facing the loss of livelihood, security and the protection provided by their families and communities. This financial and protection insecurity has resulted in families marrying their daughters at younger ages [10]. Indeed, the early marriage rates among Syrian girls between the ages of 15–17 has increased by threefold since the civil war [11, 12]. Moreover, evidence suggests that these girls’ husbands are often the primary decision makers regarding family formation, impacting their access to comprehensive SRH services [10].

Research conducted in refugee camps indicate that women and adolescents are at increased risk of physical and sexual violence and rape [13]. Findings from recent studies suggest that women and girls face harassment from both humanitarian workers and men from their community, especially during instances when women and adolescents need to travel to remote areas to purchase food and water and/or access open toilets [13, 14]. Further, a study conducted by UNWomen exploring intimate partner violence (IPV) in Jordan found that Syrian refugee women and girls are more likely to experience a form of IPV, mainly intra-family violence, in comparison to their Jordanian counterparts [10].

During the onset of the crisis in 2011, Syrian refugees in Jordan benefited from free public health services. However, this changed between 2012 to 2014, as they were required to show both their local service card and the UNHCR registration to get free access to health care. From November 2014, free health care was rescinded for Syrian refugees, but they still received the same subsidies as uninsured Jordanians. Finally, as of January 2018, the government revoked access to subsidized health care for all Syrian refugees residing outside of refugee camps [15]. The primary and secondary health care for Syrian refugees, residing in the camps, are still fully covered by the UNHCR. However, the majority of refuges are unfortunately not insured and therefore have to pay health-related fees for hospitals and clinics [16].

As estimated by UNFPA in 2015, as a result of inadequate international funding, 70,000 pregnant Syrian women had given birth in unsafe conditions due to inadequate access to maternal health services [17] and skilled birth attendants during delivery. A proportion of these women include Syrian refugee women in Jordan. Although the SRH needs of displaced and refugee populations have increased globally, funding for such programming has decreased over the last few years. For example, UNFPA in 2015 received less than half the funding it required to meet the essential SRH needs of women and adolescents [18]. This has important implications on the quality and capacity of the SRH humanitarian response and service delivery [14]. Given this context, it is evident that there still remains significant need for SRH service delivery for Syrian refugees in Jordan. The Minimum Initial Service Package (MISP) is a series of crucial actions required to respond to RH needs at the onset of every humanitarian crisis. The MISP is not solely kits of equipment and supplies; it is a set of objectives and activities that must be implemented in a coordinated manner by appropriately trained staff. This set of life-saving activities forms the starting point for ensuring quality RH service delivery in even the worst scenarios. These actions should be sustained and expanded with comprehensive RH services throughout protracted crises and recovery (See Table 1).

**Table 1:** Minimum Initial Service Package (MISP) Objectives and Activities

1. **Ensure the health sector/cluster identifies an organization to lead implementation of the MISP. The lead SRH organization:** • Nominates an SRH Coordinator to provide technical and operational support to all agencies providing health services • Hosts regular meetings with all relevant stakeholders to facilitate coordinated action to ensure implementation of the MISP • Reports back to the health cluster, GBV sub-cluster, and/or HIV national coordination meetings on any issues related to MISP implementation. • In tandem with health/GBV/HIV coordination mechanisms ensures mapping and analysis of existing SRH services • Shares information about the availability of SRH services and commodities • Ensures the community is aware of the availability and location of reproductive health services	
2. **Prevent sexual violence and respond to the needs of survivors:** • Work with other clusters especially the protection or gender-based violence sub-cluster to put in place preventative measures at community, local, and district levels including health facilities to protect affected populations, particularly women and girls, from sexual violence • Make clinical care and referral to other supportive services available for survivors of sexual violence • Put in place confidential and safe spaces within the health facilities to receive and provide survivors of sexual violence with appropriate clinical care and referral	
3. **Prevent the transmission of and reduce morbidity and mortality due to HIV and other STIs:** • Establish safe and rational use of blood transfusion • Ensure application of standard precautions • Guarantee the availability of free lubricated male condoms and, where applicable (e.g., already used by the population), ensure provision of female condoms • Support the provision of antiretrovirals (ARVs) to continue treatment for people who were enrolled in an anti-retroviral therapy (ART) program prior to the emergency, including women who were enrolled in PMTCT programs • Provide PEP to survivors of sexual violence as appropriate and for occupational exposure • Support the provision of co-trimoxazole prophylaxis for opportunistic infections for patients found to have HIV or already diagnosed with HIV • Ensure the availability in health facilities of syndromic diagnosis and treatment of STIs	
4. **Prevent Excess maternal and newborn morbidity and mortality:** • Ensure availability and accessibility of clean and safe delivery, essential newborn care, and lifesaving emergency obstetric and newborn care (EmONC) services including: • At referral hospital level: Skilled medical staff and supplies for provision of comprehensive emergency obstetric and newborn care (CEmONC) to manage • At health facility level: Skilled birth attendants and supplies for uncomplicated vaginal births and provision of basic obstetric and newborn care (BEmONC) • At community level: Provision of information to the community about the availability of safe delivery and EmONC services and the importance of seeking care from health facilities. Clean delivery kits should be provided to visibly pregnant women and birth attendants to promote clean home deliveries when access to a health facility is not possible • Establish a 24 h per day 7 days per week referral system to facilitate transport and communication from the community to the health center and hospital • Ensure the availability of life saving post-abortion care in health centers and hospitals • Ensure availability of supplies and commodities for clean delivery and immediate newborn care where access to a health facility is not possible or unreliable	
5. **Prevent unintended pregnancies:** • Ensure availability of a range of long-acting reversible and short-acting contraceptive methods (including male and female condoms and emergency contraception) at primary health care facilities to meet demand • Provide information, including existing information, education, and communications (IEC) materials, and contraceptive counseling that emphasizes informed choice and consent, effectiveness, client privacy and confidentiality, equity, and non-discrimination • Ensure the community is aware of the availability of contraceptives for women, adolescents, and men	
**Plan for comprehensive SRH services:** • Integrated into primary health care as soon as possible. • Work with the health sector/cluster partners to address the six-health system building blocks: service delivery; health workforce; health information system; medical commodities; financing; and, governance and leadership.	

Note: It is also important to ensure that safe abortion care is available, to the full extent of the law, in health centers and hospital facilities

Almost 9 years into the crisis, available documentation on the MISP implementation in Jordan and the transitioning from MISP to comprehensive SRH services is minimal and limited. The Syrian crisis has caused a widespread deterioration of the public health system, encompassing a decline in the number of health professionals and facilities to meet the needs of the host as well as the refugee population in Jordan [19]. While the evidence on the effectiveness of SRH interventions is extensive, the gaps still needed to be examined to explore why such shift of SRH interventions to comprehensive services did not happen in Jordan. Thus, it is essential to examine evidence on the effectiveness of SRH interventions in Jordan to identify gaps and provide policy briefs to decision makers that will contribute to improving SRH service delivery to both refugee and host communities.

This systematic review aims to 1) examine available published literature on the provision of the MISP for Syrian refugees in Jordan; 2) to describe and identify the current SRH issues and challenges experienced by Syrian refugee (with particular emphasis on women and girls) in Jordan; and 3) to reflect on the quality of service delivery, ability to transition to comprehensive SRH services, as well as reflect prevailing bottlenecks and opportunities impacting such transition.

## Methods

This review adopted the systematic review procedures by Khan, Kunz, Kleijnen, & Antes [20]. As the first step, the research question was stated as a free form query, which informed the three aims described above. In the second step, a search strategy was formulated using combination of search terms with Boolean operators to conduct an extensive search of multiple resources (research databases) to identify relevant work. The identified citations were narrowed down by inclusion and exclusion criteria (as provided in Table 2). In the third step, selected studies were further assessed for their quality and to explore and ensure heterogeneity of the findings. In the fourth step, collected evidence were synthesized summarized (see Table 3).

**Table 2:** Inclusion and exclusion criteria

Category	Included	Excluded
Population of interest	Crisis-affected populations receiving MISP, general SRH services and interventions in humanitarian contexts (as defined by IAWG), specifically refugee and internally displaced persons. Within the definition, this population was further limited to Syrian refugees in Jordan.	Studies that did not include Syrian populations
Health outcomes or Outputs	Primary outcomes (Prevent SV, changes in maternal mortality and morbidity rates from HIV, STI prevention, GBV, and other SRH areas) Secondary outcomes (Respond to needs of SV and GBV survivors, prevent unintended pregnancy through contraceptive use)	Studies which did not quantify the MISP and/or SRH-related health indicators.
Intervention	Any provision of the SRH services included in MISP, not only if they were provided as part of a MISP intervention	
Humanitarian crisis	Studies included during the onset of a crisis, emergency, chronic, and early recovery phases of the crisis Reports of findings by organizations involved in humanitarian contexts	Non-empirical work, such as commentaries and book reviews were excluded. Manuscripts that had not undergone peer-review, such as dissertations and conference abstracts.
Study types and Designs	All qualitative study designs or mixed methods	
Publication date	February 2011 to May 2019	
Language	English	Other languages

*Abbreviations: MISP* minimum initial service package, *IAWG* inter-agency working group, *STI* sexually transmitted infection, *SV* sexual violence, *GBV* gender-based violence, *SRH* sexual reproductive health

Search terms for SRH were based on the standardized definitions from the International Conference on Population and Development in 1994 as well as the revised 2018 IAFM (Inter-Agency Field Manual) objectives described above [21]. Sexual and reproductive health (SRH) refers to the “constellation of methods, techniques and services that contribute to reproductive health (RH) and well-being by preventing and solving RH problems”. These SRH and MISP terms were carefully explored in order to ensure that no literature has been unintentionally omitted and to ensure the search is as comprehensive and up to date as possible.

For the purpose of this systematic review, a humanitarian crisis is defined as a serious disruption of the functioning of a community or a society causing widespread human, material, economic or environmental losses which exceeds the ability of the affected community/society to cope with its own resources, thus necessitating a request to national and international aid and assistants [22].

This review included both peer-reviewed articles and grey literature reports. The dates of publications were restricted to 1 January 2011–30 November 2019. We searched for peer-review published literature across three databases: PubMed, Medline (Ovid), and Scopus. For the grey literature, websites of the leading international agencies providing humanitarian support were explored, such as: United Nations Population Fund (UNFPA), GirlsnotBrides, Raise Initiative, Inter-Agency Working Group on Reproductive Health in Crises (IAWG), Save the children, the International Rescue Committee (IRC), CARE, International Committee of the Red Cross (ICRC), International Planned Parenthood Federation (IPPF) and UNWomen. Google search engine was used to identify relevant literature and reports not published in peer-reviewed journals. Further, the search was supplemented by screening the references cited by the eligibly included papers to identify potentially missed peer-reviewed articles.

Electronic databases were searched for both free text key words and Medical Subject Headings (MeSH) to identify all relevant terms. Boolean operator ‘OR’ was used to widen search to include free text keywords and MeSH terms defining the same concepts, while ‘AND’ was used to narrow down the search by combining different concepts into the search strategy and arrive at the final search results.

We used key search terms (‘Sexual and Reproductive Health’ OR ‘SRH’ OR ‘Reproductive Health’ OR ‘RH’ OR ‘Sexual Violence’ OR ‘Family violence’ OR Intimate partner violence’ OR ‘IPV’ OR ‘(Sexual and) Gender-based Violence’, OR ‘(S)GBV’, OR ‘Minimum Initial Service Package’, OR ‘MISP’), AND (‘Women’, OR ‘Girls’, OR ‘Adolescents’), AND (‘Syrian’ OR ‘Refugee’, OR ‘Jordan’), AND (‘Humanitarian crisis’, OR ‘War’, OR ‘(Armed) conflict’ OR ‘Crisis’ OR ‘Disaster’). Further, we used star truncation (*), as needed, to account for multiple endings of terms, such as “Adolescent*” for adolescent, adolescents, and adolescence. We also applied search filters to limit published article and reports between 2011— 2019.

Inclusion and exclusion criteria were based on the Inter-Agency Field Manual (IAFM) on reproductive health in humanitarian settings [23]; these are outlined in Table 2.

**Fig. 1:**
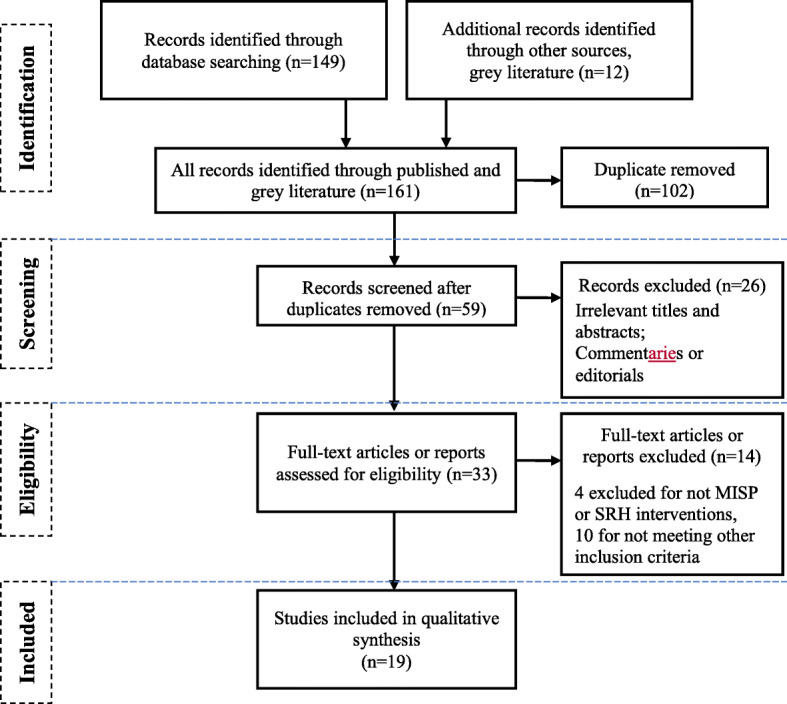
Adapted the Preferred Reporting Items for Systematic Reviews and Meta-Analyses (PRISMA [24]) flow chart

Two researchers independently downloaded all identified citations into a Mendeley library, and each assessor applied the standard data-screening process as shown in the PRISMA flow chart [33] in Fig. 1. Initially, 161 records were identified: 149 through the research databases and 12 through other sources (grey literature). A total of 102 records were excluded as duplicates, leaving us with 59 records. In the screening stage, the titles and abstracts for all 59 records were reviewed. A total of 26 more records were excluded, because they either had irrelevant titles and abstract or they were commentaries or editorials. Thus, a total of 33 full-text articles or reports were included to be assessed against eligibility criteria (see Table 2). During this stage, 14 full-text articles or reports were excluded: 4 were excluded because they were not MISP or SRH interventions; and 10 were excluded for not meeting other inclusion criteria. Finally, a total of 19 papers (published articles or reports) were included in our qualitative synthesis, as shown in the PRISMA flow chart below. Following the title, abstract and full text screening and review, the needed information from the selected studies and reports were extracted into an Excel database (available as a table at the ‘Results’ section of this paper and as a separate Excel sheet). The extraction focused on the following data: study design and methods, participant characteristics, research settings, health outcomes, MISP intervention description, key findings and recommendations.

## Results

Out of 19 studies included in our review, 11 were peer-reviewed articles and 8 were reports or other types of research papers. Although, the literature review focused on all publications between 2011 and 2019, all studies that met the inclusion criteria were conducted and published post-2013.

In line with the MISP objectives, we, here in discuss the overall results of this review as per the following key areas: RH coordination, barriers to MISP/SRH services (which also covers MISP implementation), gender-based violence and sexual violence, maternal health services, family planning, and HIV/STI prevention (See Table 3).

**Table 3:** Identified Peer-Reviewed Articles and Reports

Author(s)/ Year	Title	Type of Publication	Journal or Agency	Design/ Methods	Major Findings	Discussions, Implications, Recommendations
Gausman J et al., 2019 [25]	How do Jordanian and Syrian youth living in Jordan envision their sexual and reproductive health needs? A concept mapping study protocol	Peer-reviewed article	BMJ Open	Concept mapping	Other studies indicate that Jordanian youth require reproductive health-related support, information and services; however, there remains very limited data as to how youth envision their SRH challenges and needs. Findings of this study will be released in future.	Many of the SRH issues are inter-related and interact with each other. Recommendations will be released in future.
Al-Rousan T et al., 2018 [16]	Health needs and priorities of Syrian refugees in camps and urban settings in Jordan: perspectives of refugees and health care providers	Peer-reviewed article	EMHJ	Qualitative and quantitative methodologies.	Many different problems were revealed such as cost, limited resources, changing policies, livelihoods and poor health literacy impeded delivery of public and clinical health services. Of note is that, according to health care providers and key informants, Syrian refugees primarily seek health care for acute conditions, including respiratory illness, fever, diarrhea and injuries. Providers noted that the primary reason for reduced access to antenatal care was the lack of female physicians.	Syrian refugees identified cost as the main barrier to health care access. Both refugees and health care providers emphasized the importance of directing more resources to chronic diseases and mental health One of the recommendations was to ensure availability of female physicians to provide these services in a culturally sensitive manner
UNHCR, 2018 [3]	Health access and utilization survey: Access to healthcare services among Syrian refugees in Jordan	Report	UNHCR	Cross-sectional Survey	Women who needed antenatal care (ANC) were 17% of WRA or women of reproductive age (15–49 years), while 90% of the pregnant females received ANC during the last 2 years. Percentage of pregnant women who had difficulty accessing ANC was 16%. The highest number of pregnant females faced difficulties in user fees (55%) and transport cost (23%) in 2018, which were less in 2017 (46 and 19% respectively). An increase in child deliveries was witnessed among Syrian households in 2018, with 86% females delivering newborns compared to 74% in 2017. Pregnant females with more than 4 ANC visits represent 67.9%. Deliveries location are divided among governmental hospitals (48%) and private clinics/hospitals (46%). Healthcare services were needed by 49% of household members in 2018 compared to 37% in 2017.	
Asaf Y, 2017 [14]	Syrian Women and the Refugee Crisis: Surviving the Conflict, Building Peace, and Taking New Gender Roles	Peer-reviewed article	Social Sciences	Secondary research, review	Refugees who are not registered or those living outside of camps have more difficulty to public services, They also have no right to work in Jordan, Turkey, and Lebanon. Gender is not mainstreamed in policies, affecting women and girls in particular. Women are targeted with various forms of gender-based violence, while the issues they raise are often marginalized or excluded.	
DeJong J et al., 2017 [19]	Reproductive, maternal, neonatal and child health in conflict: a case study on Syria using countdown indicators	Peer-reviewed article	BMJ Global Health	Systematic literature review	Coverage rates of most key evidence-based interventions in reproductive, maternal, newborn and child health declined in Syria: among refugees in neighboring countries the picture was more mixed as compared with pre-conflict Syria. And in conflict settings such as that of Syria, coverage rates of such interventions are often unknown or difficult to ascertain.	Research, monitoring and evaluation in humanitarian settings could better inform public health interventions if findings were more widely shared, methodologies were more explicit and globally agreed definitions and indicators were used consistently.
Samari, G., 2017 [26]	Syrian Refugees Women’s Health in Lebanon, Turkey, Jordan and Recommendations for Improved Practice	Peer-reviewed article	World Medical Health Policy	Literature review	Sexual and gender-based violence was associated with a reduced use of modern contraceptives. Menstrual irregularity, unplanned pregnancies, preterm birth, and infant morbidity are ongoing issues. It was also noted that increased availability of female physicians will provide women’s health services in a culturally appropriate manner.	Taking a multilevel approach to eliminate social and service delivery barriers that prevent access to care, conducting thorough needs assessments, and creating policy and programmatic solutions that establish long term care for Syrian refugee women. One of the recommendations was also to ensure availability of female physicians to provide these services in a culturally sensitive manner
UNHCR, 2017 [27]	Health access and utilization survey: Access to health services in Jordan among Syrian refugees	Report	UNHCR	Cross-sectional Survey	An increase in pregnant females who received antenatal care (88% vs. 85% in year 2016). A significant increase in difficulty accessing ANC, and an increase in those who can’t afford fees or transport compared to 2016. A decrease in the deliveries free of cost. Majority of deliveries took place mainly in governmental (53%) and private facilities (40%).	
Ay et al., 2016 [28]	The perceived barriers of access to health care among a group of non-camp Syrian refugees in Jordan	Peer-reviewed article	International Journal of Health Services	Cross-sectional, analytical, observational study using convenience and snowball sampling for data collection	Preventive and primary health care were more accessible than advanced services. Structural and financial barriers hindered access.	The capacities of health facilities at different levels should be increased. Enhanced information sharing among health providers can improve identification of needs and gaps.
Clark et al., 2016 [7]	The influence of family violence and child marriage on unmet need for family planning in Jordan	Peer-reviewed article	Journal of Family Planning and Reproductive Health Care	Logistic regression	Experiencing family and intimate partner violence (IPV) has a compounding effect on unmet need for contraception among women who married as minors. Women married as minors who experienced IPV and FV had a four-fold higher likelihood of having an unmet need compared to those experiencing only IPV. No interaction between IPV and FV was detected for women married at or above majority.	Screening for intimate partner violence (IPV) and family violence in health services may identify women who are especially vulnerable to having an unmet need for contraception. Laws that prohibit child marriage should be strengthened and health sector screening for violence experience could help identify women at risk of unmet need and improve their reproductive agency.
JCAP, 2016 [11]	Family Planning Among Syrian Refugees in Jordan	Report	USAID/ JCAP	Desk review	Majority (85%) of Syrian refugees live outside camps, most of them ‘extremely vulnerable.’ Use of modern methods was lower among Syrians, at 22% vs. 29% of Jordanians. In 2015 only 64% of households of registered Syrian refugees knew that refugees had subsidized access to government primary health centers. While early marriage has long been an accepted practice in Syria, registered marriages among Syrian refugees including girls aged 15–17 increased almost threefold, from 12% in 2011 to nearly 32% in 2014. There is no reliable information on the number or proportion of Syrian refugees in Jordan who have experienced GBV Many Syrian refugees living outside of camps are unaware of health services available to them, especially women who have limited mobility.	Support the national health system and services to strengthen the quality and accessibility of comprehensive RH/FP information and services for all residents in Jordan including Syrian refugees. Increase Syrian refugee access to accurate information about reproductive health and available services. Ensure broad awareness of the MOH policy issued in February 2016 regarding free maternal and health and family planning services in public health facilities for registered Syrian refugees. Empower girls and educate parents and community members against child marriage. Prevent early pregnancy among young married girls through counseling. Increase access to accurate information about FV, IPV and GBV.
Juraibei, et al., 2016 [12]	Reproductive Health Services for Syrians Living Outside Camps in Jordan	Report	The Higher Population Council	Cross-sectional, descriptive study of SRH service recipients and providers	The majority of organizations providing RH services to non-camp Syrians refugees reported financial challenges and high operational costs and costs incurred by Syrians reach centers, socially-sanctioned barriers to providing Syrians with RH services, a general lack of awareness, family intervention in personal healthcare choices, restrictive norms/ traditions, and early marriage. 68% receiving RH services were females aged 12–49 years and half of them receiving these services from NGOs. 41.4% used modern contraceptive methods. They had medium levels of satisfaction with the received RH services. Most of those not going to RH centers noted far location from their residence, overcrowded, lack of medical specializations, and poor treatment by center workers. Also, new security cards were one big public policy barriers to Syrians’ access to RH clinics.	Raising awareness among Syrians residing outside of refugee camps on RH/FP services, early marriage, and pregnancy spacing. Provide financial support to MOH and technical/ logistical support to organizations providing RH services to non-camp Syrian refugees. Develop employee standards at RH clinics. Continuously monitor organizations providing RH services to non-camp Syrian refugees to ensure quality services and efficient operations. Activate legislation related to early marriage. Reassess ID card policies so as to allow cardholders to receive treatment at any health center outside of their residential areas.
Smith, H., 2016 [29]	Syrian refugee women in Jordan: Family Planning Preferences and Barriers in a Host Community	Peer-reviewed article	SIT Digital Collection	Cross-sectional study	Although 71% of the women surveyed found their RH care provider to be extremely or somewhat trustworthy, nearly 86% of them said their health care provider did not initiate a conversation about birth control, causing a lack of awareness. Only 36% of women surveyed had attended an informal support group. Many women interviewed stated that birth control was harder to access in Jordan than in Syria due to lack of affordability and health insurance.	This study recommends that birth control be more prevalent and discussed more in reproductive health clinics while being made more affordable.
West et al., 2017 [30]	Factors in use of family planning services by Syrian women in a refugee camp in Jordan	Peer-reviewed article	Family Planning and Reproductive Health Care	A small-scale qualitative study	Family planning (FP) services were available; however, barriers to service uptake included poor awareness of FP services, overburdened health services, cultural pressures regarding fertility, and poorly trained service providers. International attempts to address refugees’ family planning needs remain inconsistent.	Future research is needed into effective methods for international dissemination of evidence for established interventions in FP and how to adapt such interventions in different refugee settings.
Essaid M et al., 2015 [13]	Gender Based Violence Against Women and Girls Displaced by the Syrian Conflict in South Lebanon and North Jordan: Scope of Violence and health correlates	Research paper	Spanish Agency for International Development Cooperation	Mixed-methods design	GBV is a significant problem in North Jordan and South Lebanon for Syrian refugee women and girls and frequently restrict their movement, causes them mental and physical distress, and occurs both inside and outside of the home. GBV is a contributor to poor RH outcomes among Syrian refugee women and highlights the risk to women’s reproductive and sexual health. Barriers to support seeking included shame because revealing family violence is perceived as a violation of social norms, fear of the consequences, lack of trust in service providers, and inability to leave the home due to lack of finances or childcare.	Integrate the updated Inter-Agency Standing Committee (IASC) guidelines into GBV response in Lebanon and Jordan.
Higher Population Council King Hussein Foundation, 2015 [31]	Jordan Agenda Setting for Sexual and Reproductive Health and Rights Knowledge Platform	Report	**Higher Population Council King Hussein Foundation** Share-Net International	Agenda-setting discussion	Improved SRHR can result from: better information and greater freedom of choice for young people about their sexuality; improved access to reproductive health commodities; better sexual and reproductive health care (during pregnancy and childbirth, including safe abortion); and, greater respect for the sexual and reproductive rights of groups who are currently denied these rights.	
Krause et al., 2015 [4]	Reproductive health services for Syrian refugees in Zaatari Camp and Irbid city, Hashemite Kingdom of Jordan: An evaluation of the Minimum Initial Services Package.	Peer-reviewed article	Conflict and Health	Formative evaluation approach	Lead health agencies addressed the MISP by securing funding and supplies and establishing RH focal points, services and coordination mechanisms. However, Irbid City was less likely to be included in coordination activities and health facilities reported challenges in human resource capacity. Access to clinical management of rape survivors was limited, and both women and service provider’s knowledge about availability of these services was low. Activities to reduce the transmission of HIV and to prevent excess maternal and newborn morbidity and mortality were available, although some interventions needed strengthening. Some planning for comprehensive RH services, including health indicator collection, was delayed. Contraceptives were available to meet demand. Syndromic treatment of sexually transmitted infections and antiretrovirals for continuing users were not available. In general refugee women and adolescent girls perceived clinical services negatively and complained about the lack of necessities.	MISP services and key elements to support implementation were largely in place. Pre-existing Jordanian health infrastructure, prior MISP trainings, dedicated leadership and available funding and supplies facilitated MISP implementation. The lack of a national protocol on clinical management of rape survivors hindered provision of these services, while communities’ lack of information about the health benefits of the services as well as perceived cultural repercussions likely contributed to no recent service uptake from survivors. This information can inform MISP programming in this setting.
Masterson A et al., 2014 [32]	Assessment of reproductive health and violence against women among displaced Syrians in Lebanon	Peer-reviewed article	BMC Women’s Health	Cross-sectional needs assessment, Survey, Bivariate and multivariate analyses	We interviewed 452 Syrian refugee women ages 18–45 who had been in Lebanon for an average of 5.1 (± 3.7) months. Reported gynecologic conditions were common. Of women who experienced conflict-related violence (30.8%) and non-partner sexual violence (3.1%), the majority did not seek medical care (64.6%). Conflict violence and stress score was significantly associated with reported gynecologic conditions, and stress score was found to mediate the relationship between exposure to conflict violence and self-rated health.	This study contributes to the understanding of experience of conflict violence among women, stress, and reproductive health needs. Findings demonstrate the need for better targeting of reproductive health services in refugee settings, as well as referral to psychosocial services for survivors of violence. Although not a main recommendation, increased availability of female physicians will provide women’s health services in a culturally appropriate manner.
Doedens W et al., 2013 [33]	Reproductive Health Services for Syrian Refugees in Zaatri Refugee Camp and Irbid City, Jordan.	Report	Washington, DC: US Department of State, Bureau of Population, Refugees and Migration	Mixed quantitative and qualitative methods	In spite of the steady influx of refugees into Jordan that has strained the resource capacity of this humanitarian emergency response, the agencies that provide RH services have been able to implement the MISP for the most part, although there is need for some key improvements. In this setting, the study team found some challenges, such as balancing the increasing demands for services while maintaining quality and managing information flow among multiple stakeholders. It is vital to stay informed and listen to the needs of Syrian refugees in Jordan to improve RH outcomes in the months to come. Key informants were aware of the five MISP objectives. However, there was very limited understanding of the additional priorities of the MISP such as ensuring contraceptives are available to meet the demand; treatment for sexually transmitted infections (STIs) is available to people presenting with symptoms; antiretrovirals (ARV) are available to current users; and menstrual, hygiene supplies are available. A number of key elements to support implementation of the MISP were in place, including a dedicated lead agency to support MISP implementation within the health sector, a focal point for RH coordination, regular RH coordination meetings, and RH kits and supplies, and funding for MISP implementation. However, key informants reported that RH coordination was insufficient for the urban areas; not all key stakeholders participated in coordination; protocols for care for survivors of sexual violence were incomplete or STIs did not exist; and that key informants would like UNFPA to share the information that it collects from stakeholders among stakeholders. Syrian refugee women discussed security fears that they had in relation to using the latrines at night due to a lack of lighting. While services existed to manage sexual violence (SV), they were limited, and community and provider knowledge of the services was low. In terms of additional priorities to the MISP, modern methods of family planning was available (although condom distribution limited), syndromic treatment for people presenting with symptoms of STIs was not available, the situation of continuing ARVs for refugees already on ARVs was unknown and menstrual hygiene supplies were insufficient. MISP contingency plans were established but not activated. Jordan has undertaken some activities on disaster risk reduction although it was unclear if there have been initiatives to address health and RH. Barriers to MISP implementation included a lack of adequate staffing in urban areas and of clear RH protocols, particularly on care for survivors of SV, and management of STIs); less focus by the RH working on urban populations compared with the camp population; and lack of capacity to implement the MISP contingency plan.	Strengthen coordination to address the RH needs of urban refugee populations; facilitate the participation of key stakeholders; address RH protocols, particularly, finalize the clinical care for SV survivors protocol; identify STIs management and protocols for referral of and caring for person living with HIV (PLHIV); improve data collection and use of data for action; and support information, education and communication (IEC) campaigns on the benefits to seeking care and the availability, location and hours of services in both urban areas and Zaatri refugee camp. Improve free condom distribution with sensitivity to cultural norms. Scale up the availability of clinical care for survivors of SV at service delivery sites and consider integrating the protocol into the Family Protection Department where forensic doctors are available and could be trained. Strengthen community outreach, participation and services along with information and education, including for adolescents and people with disabilities, by utilizing existing IEC campaign resource materials on the MISP and family planning, and ensuring all service delivery is physically accessible and inclusive of people with disabilities. Improve the health care environment with adequate staffing, particularly female doctors and by addressing the interactions between health care providers and Syrians so that Syrian women feel comfortable while seeking care. Advocate for Syrian health care providers to be involved in providing health care services to the refugees.
UN Women. (2013)	Gender-based violence and child protection among Syrian refugees in Jordan, with a focus on early marriage: Inter-agency assessment	Report	United Nations Entity for Gender Equality and the Empowerment of Women (UN Women)	Mixed method: Cross-sectional survey, FGDs, and KI interviews	Women and children (80% of Jordan’s Syrian refugee population) are vulnerable to an increased risk of sexual, physical, and psychological abuse, yet have limited opportunities to access safe spaces or social services. Among those girls who were employed, 80% work in either domestic work or agriculture, both of which are known to be high-risk sectors for physical abuse and sexual exploitation. Gender-based Violence (GBV) remains a private and sensitive issue that is largely addressed within the home setting. Specialized, confidential, and supportive services currently available to Syrian women and children survivors of GBV are not sufficient, and when such resources are available, Syrian refugees are very often (83%) unaware of them. Early Marriage is a common experience for Syrian girls (51.3% married before the age of 18 and most prior to their arrival in Jordan), yet women of all ages knew someone who had experienced early marriage. With unemployment and dwindling family resources, more prolonged displacement will lead to greater likelihood of early marriage for girls, while many acknowledged heading households and rearing children at such a young age to be stressful and challenging.	Expand and improve services to respond to GBV including access to sexual and reproductive health information and programs. Increase access to safe spaces in which trained professionals can provide psychosocial support for Syrian refugees who are experiencing violence, abuse, and exploitation. Increase availability of mobile clinics/units including GBV services to enable outreach in remote areas. Donors should allocate predictable funding and sustain funding to support implementation of safe, non-stigmatizing, culturally relevant initiatives to prevent and respond to GBV strategies that will have longer-term impact on refugees, promoting durable solutions for “the day after” in Syria. GBV interventions should prioritize participation of men and boys as part of a comprehensive strategy to prevent, mitigate and respond to related risks. Livelihood programs should be scaled up to support resilience and positive coping mechanisms of Syrian refugees to support broader prevention and reduction of GBV.

### Coordination of RH services

The results show that although all studies and reports found eligible for this review discussed certain SRH services, only three peer-reviewed articles [4, 26, 30] and two reports [29, 33] discussed MISP as a package with focus on selected MISP objectives. In addition, one 2013 study in the form of a report [33], which was also published in 2015 [4], focused on evaluating MISP delivery in both camp and non-camp settings. This study examined agencies providing RH services, it showed that although many of these agencies were able to implement the MISP, key improvements were still needed. It was noted that funding, supplies, and RH lead focal points responsible for services’ provision and coordination mechanisms were institutionalized mainly in camp settings, while it was more problematic for set up in non-camp/urban areas. Many factors were attributed to this, including the limitations of effective coordination activities, growing health needs vis a vis the limited human resource capacities. Important areas that were commonly neglected and more challenging included: access to clinical management of rape survivors coupled with limited women’s and service providers’ knowledge about availability of these services, activities to reduce the transmission of HIV and to prevent excess maternal and newborn morbidity and mortality (while available required more strengthening), and finally planning for effective transition to comprehensive RH services. Contraceptives were available to meet demand, while syndromic treatment of sexually transmitted infections and antiretrovirals for continuing users were not available.

In general, based on the results of this review, Syrian refugee women and adolescent girls perceived clinical services negatively and complained about the lack of necessities. Barriers to MISP implementation included a lack of adequate staffing in urban areas and of clear RH protocols, particularly on care for survivors of sexual violence, and management of STIs; less focus by the RH working on urban populations compared with the camp population; and lack of capacity to implement the MISP contingency plan [4, 33]. Assessments of MISP have indicated mixed success with gaps in implementation, poor overall coordination, lack of donor support, lack of procedures in place, poor quality and availability of referral services, and inadequate monitoring of service delivery, lack of trained staff to prevent maternal morbidity and mortality, sexual violence, and HIV [4, 26, 29], and a lack of female staff acting as a barrier to FP access for Syrians [4, 30]. Coordination issues have been addressed in meetings hosted in Amman, but many of the solutions to improve coordination have been focused on Za’atari refugee camp as opposed for these refugees who are dispersed throughout the host communities and who make up the majority of this population [4, 29].

### Barriers to MISP/SRH services

Five peer-reviewed articles [4, 16, 26, 28, 29] and six reports [3, 11, 12, 27, 31, 33] discussed Syrian refuges access to health facilities and services in general and SRH services in particular.

A number of problems were highlighted including cost, limited resources, changing policies, livelihoods and poor health literacy. These barriers impeded delivery of public and clinical health services in both in-camp and urban settings. Syrian refugees identified cost as the main barrier to health care access [16, 29, 31]. For example, among pregnant Syrian women refugees in 2018, cost of services was reported as the leading cause limiting their access to services (reported by 55%), this was followed by incurred transportation cost (reported by 23%). These costs complaints were also reported in 2017, as 46% mentioned service costs and 19% mentioned transportation costs as barrier to access SRH services [3, 27]. For non-camp refugees, preventive and primary health care were more accessible than advanced/tertiary care services and structural and financial barriers hindered access [28]. Specialized, confidential, and counseling and other support services available to Syrian women and children survivors of SGBV (Sexual and Gender-based Violence) were not sufficient, and—when available—Syrian refugees were very often (83%) unaware of them [10]. According to one study in 2015, only 64% of households of registered Syrian refugees knew that refugees had subsidized access to government primary health centers [11]. In one qualitative study, focus group participants indicated that the new security cards were one of the recent greatest imposed public policy restrictions preventing Syrians’ access to RH clinics [12].

Refugees who are not eligible to receive primary health care mainly resort to private sectors, which are often perceived by them as highly expensive and unaffordable. A similar trend is observed when it comes to seek medication access, only 10% of Syrian refugees refer to pharmacies as compared to 18% who depend on international NGOs institutions as a source of health care and medication use [34].

### Sexual and Gender-Based Violence (SGBV)

Five peer-reviewed articles [7, 14, 26, 32, 33] and five reports [10–13, 33] discussed issues surrounding gender-based violence (GBV) and sexual violence (SV).

Limited reliable information exists on the numbers or proportions of Syrian refugees in Jordan who have experienced SGBV. However, it is widely acknowledged, in most of the above-cited sources, that the conflict in Syria and the displacement of Syrians to Jordan has exposed women and children to increased risk of SGBV, abuse, neglect, exploitation and other forms of violence (including early and forced marriage). Women face various forms of violence and they are often marginalized or not effectively assisted (by family, friends, and/or health-care providers) to seek the needed care. The cultural belief system act as one of the main barriers preventing for example adolescents, who experience early marriage and transactional sex, to seek help when needed [13]. As seeking help will further increase the risks of imposed violence often leading to catastrophic consequences such as leading to becoming disowned by their family members or to exposing them to even worse acts such as “honor killing” [4, 13, 33].

One study reported SGBV to be a significant problem among Syrian refugees who are residing in either North Jordan or South Lebanon [13]. These Syrian refugee women and girls are compounded by their inability to seek help for SGBV because of severe restrictions on their movement. Their inability to seek the needed help is often exposing them to mental and physical distress. Barriers observed to seek help or support included: shame because revealing family violence is perceived as a violation of social norms, fear of the consequences, lack of trust in service providers, and inability to leave the home due to lack of finances or childcare [13]. Another study on women refugees, who experienced conflict-related violence (30.8%) and non-partner sexual violence (3.1%), found that the majority did not seek medical care (64.6%) [32]. Thus, SGBV remains a private and sensitive issue that is largely addressed within the home setting. Access to clinical management of rape survivors was limited, and both women and service providers’ knowledge about availability of such services was low [4, 33]. Also, specialized, confidential, and supportive services available to Syrian women and children survivors of SGBV are not sufficient, and Syrian refugees are generally unaware of services available for SGBV.

Early marriage has been frequently registered among Syrian refugees in Jordan since the crisis. According to the report by the Jordan Communication, Advocacy and Policy Activity (JCAP) project, although child marriage (between 15 and 17) is a generally accepted practice in Syria, rates of registered child marriages among Syrian refugees for girls aged 15–17 has increased by almost threefold in Jordan, from 12% in 2011 to nearly 32% in 2014 [11, 12]. According to earlier data from Syria prior to the crisis, it is estimated that around 51.3% of young girls married before the age of 18, i.e. prior to refuge into Jordan. Moreover, women of all ages knew someone who had experienced early marriage. The report indicates factor contributing to these increased rates are associated with unemployment and dwindling family resources as well as prolonged displacement [10]. Syrian women refugees who are unaccompanied by a male family member often marry their daughters as young as 13 or 15 years of age, as they perceive that this could protect them from being assaulted and/or facing hardships including financial hardships; they believe this will provide with ‘roof over their heads’. Further, it is estimated that around 48% of these girls have been married off to men at least 10 years older. This often increases the vulnerabilities of these young girls to domestic abuse, poverty, and health problems [14].

SGBV poses significant SRH impacts, as it is directly associated with poorer SRH outcomes among Syrian refugee women. One review found that sexual and gender-based violence was associated with a reduced use of modern contraceptives [26]. Similarly, evidence from another study showed that experiencing family violence (FV) and intimate partner violence (IPV) had a compounding effect on unmet need for contraception among women who were married as minors. Women married as minors (child marriage) who experienced IPV and FV had a four-fold higher likelihood of having an unmet family planning needs compared to those experiencing only IPV. No interaction between IPV and FV was detected for women married at or above majority [7]. This study used logistic regression to test whether IPV and FV were independently associated with unmet need by age at marriage.

Moreover, in Jordan, management of the consequences of sexual violence was found of limited quality. Clinical protocols of care informing practitioners on how to best manage survivors of sexual violence were either lacking or incomplete [33].

This is also true for access to emergency contraception (EC) [11]. Although MOH and UNFPA have increased their efforts to improve clinical care for sexual assault survivors as well as IPV survivors through the distribution of clinical management of rape (CMR) kits to health clinics; these seem to not be used effectively as providers’ knowledge continues to be limited [11]. One study conducted in 2013 among Syrian refugees in camps found evidence of both severe supply and demand side barriers for EC and CMR. These included: providers’ biases in offering women contraception, limited knowledge among women of where to obtain SRH services, providers’ refusal to provide emergency contraception post-rape, increased rates of reported unsafe self-induced abortion. Along these lines, it has been reported that providers tend to refuse to give emergency contraception to a rape survivor and/or an unmarried woman [4, 33].

### Maternal health services

Three published studies [4, 19, 32] and one report [33] discussed maternal health services, while two reports by UNHCR [3, 27] discussed antenatal care in particular.

According to earlier assessments, activities to prevent excess maternal and newborn morbidity and mortality were available, although some interventions needed strengthening [4, 33]. According to UNHCR Health Access and Utilization Surveys in Jordan in 2017 and 2018, it has been estimated that 17% of Syrian females in reproductive age (15–49 years) needed ANC, of which 90% of them received this care. The percentage of the pregnant women who had difficulty accessing ANC was 16%. The immediate barriers to accessing care included the increased cost of use (55%) and transport cost (23%) in 2018, which were less in 2017 (46 and 19% respectively). Only 67.9% of pregnant females had 4 or more ANC visits during the course of their pregnancy. Women delivered in either governmental hospitals (48%) or private clinics/hospitals (46%) [3, 27]. ANC coverage among Syrian refugees (at least one ANC visit) in Jordan remains at similar levels to that during the pre-conflict Syria (87.7%), whereas use of at least four ANC visits has substantially dropped, especially among the non-camp Syrian refugee population. The most reported reason for this lower use was attributed to the limited availability of female physicians [16]. Within the camps in Jordan, several specialized RH facilities have been established; for this reason, postnatal care is often higher (around 50%) compared to the rates during the pre-conflict Syria. Increases in C-section rates among refugees is not prevailing practice since the conflict began. Rates of skilled birth attendance at delivery among the Syrian refugee population living in camps remain almost universal likely as a result of special provisions by UNHCR [19].

### Family planning

In addition to five eligible reports discussing family planning services coverage [3, 11, 12, 27, 33], nine peer-reviewed articles [4, 7, 13, 19, 26, 28, 29, 32, 35] examined family planning. These studies (*n* = 14) mainly examined the extent of family planning knowledge and use with specific focus on contraceptive utilization, service delivery and uptake among Syrian refugees in Jordan. The overall findings of these studies suggested that adequate family planning services are available in Jordan for Syrian refugees; yet, significant misconceptions prevail among users pertaining to the different contraceptive methods. A study conducted by UN Women [10] highlighted that this was especially true of child brides where these girls are under extreme pressure to prove their fertility. Among the common misconceptions in the community, which was perpetuated by physicians, is that IUD cannot be inserted until the girl or woman has had at least 2–3 pregnancies. Also, many girls reported overarching fears that using different forms of contraceptives could potentially lead to infertility, this was especially cited when asked about oral contraceptive pills [36]. According to one study in Al-Za’atari camp, it was found that women and girls often lack awareness and knowledge of the different family planning methods and their side effects [4]. Of the reported sides effects among women pertaining to use of family planning were anger, weight gain and the pill “weighing on their bodies”. Women of these cited misconceptions around family planning were often found to have lower levels of awareness and knowledge about prevailing workshops/support groups held for females in their communities [33]. One study in 2016 found that family and intimate partner violence (IPV) was associated with compounding effect on unmet need for contraception among women who were married as minors. This study showed that women married as minors who experienced IPV and FV had a four-fold higher likelihood of having an unmet family planning needs compared to those experiencing only IPV [7].

### HIV and STI prevention

Of the eligible included literature in this review, two articles [4, 26] and one report [33] discussed issues related to HIV and STI prevention. These papers mainly indicated inadequate STI and HIV services provided to Syrian women refugees. Other highlights included limited knowledge of the Syrian women refugees on how to reduce HIV transmission. Women associated the notion that HIV would lead to AIDS; however, their knowledge of HIV transmission methods, treatments and lifespan were very poor. The women believed that contracting HIV from blood banks was the main source of transmission as opposed to that from sexual intercourse. It was also indicated that clinics in Amman had limited resources to provide post-exposure treatments. Although male condoms were available in both clinics and in the safe female places, female condoms were neither stocked nor available. Further, health care providers were less likely to provide condoms to unmarried women. This was perceived to increase the risk of contracting HIV and other potential STIs among these refugee women population. Equally important to note is that Syrian refugees who are HIV positive are at higher risk of getting deported, which further limits the availability of HIV testing treatment among Syrian refugees in Jordan [4, 26, 33].

## Discussion

This, to our knowledge, is the first systematic review summarizing the evidence of the provision of the MISP in the humanitarian context of Jordan. Our review suggests that although Jordan has witnessed significant improvements in the national provision of SRH services for its population, there still remain gaps in the SRH service delivery for Syrian refugees in Jordan [11, 28]. The findings from our review suggest that Syrian refugee women and girls experience a wide array of barriers in accessing SRH services, including, but not limited, to limited autonomy, mobility, geographical distance, knowledge, cost and social standing [11]. We identified no studies that evaluated the utilization of interventions focused on STI and HIV treatment and prevention, post-abortion and safe abortion care.

To our knowledge, no data exists on the experiences of Syrian refugee women with HIV, which is understandable, as being HIV positive can lead to deportation from Jordan [26, 33]. Such policies are also leading to lack of limited HIV testing and treatment for Syrian refugees who are at risk of or suffering from HIV in Jordan. Thus, unmet SRH needs constitute a significant problem among Syrian women and adolescent refugees in Jordan. Syrian women and girls in Jordan also lack the adequate knowledge and tools to address their needs. As a result, huge misconceptions around FP services, FP needs, and birth spacing exist among this vulnerable population. Except for one study in 2013, that indicated many supply and demand side barriers to emergency contraception (EC) [33], no data exist on availability and use of EC among Syrian refugee women in Jordan. Furthermore, there is limited data on the overall use/ uptake rates of RH services, antenatal care, STI treatment, HIV treatment, psycho-social services, and SGBV treatment to Syrian refugees residing outside camps.

This is of grave concern, given the increased vulnerability of these displaced women and girls on one hand and the inability to inform policies on their actual needs. This affects the availability and quality of provided SRH services. The findings of this review necessitate the need for more responsive health clinics. Additionally, there is a call for international organizations and national NGOs to better deliver on the MISP objectives for the prevention of HIV transmission, by improving the delivery of HIV treatments, comprehensively managing sexual violence, as well exerting better efforts in the delivery of FP services and especially EC. More efforts are also needed to reduce maternal mortality/morbidity by addressing the huge disparities often complicating access to antenatal care for non-camp-based settings where these refugee women reside.

Based on the results, the provision and quality of SRH services provided to Syrian refugees in Jordan need to be improved. Additionally, serious efforts need to be made to enable a comprehensive delivery of SRH services, especially to women residing in non-camp-based settings and poor urban settlements. The few studies that addressed MISP interventions showed that MISP objectives were not equally implemented across the different governorates in Jordan where the refugee population cluster most. Also, effective coordination of SRH services poses real challenges to service providers. For example, SRH service providers may experience life threats associated with stigma and cultural taboos about reproductive and sexual health; they may also experience legal and policy constraints related to the age and marital status of individuals seeking SRH services. Moreover, lack of human resources capacity, sustainable training programs, knowledge about SRH services represented equally important impediments that require immediate attention [4].

Finally, this review provided a contextual understanding of the status of the MISP implementation by governmental, local and international agencies in Jordan. It also provided a thoughtful recognition of the Syrian women/girls’ knowledge and attitudes towards SRH.

### Limitations

The authors of this paper have identified multiple limitations to this systematic review. One of them is lack of bibliographic database on non-published reports from civil societies and non-governmental organizations. This may imply that studies conducted by these organizations were missed. Similarly, studies about SRH interventions that did not explicitly refer to the MISP and SRH interventions in the key words, title, or abstract may not have been identified. The search may have also missed titles and abstracts in languages other than English. Screening by two team members may have resulted in varied application of the criteria; however, since the two team members shared the same location, this enabled constant discussion and comparison between them and allowed for a 98% inter-rater reliability. The cross-sectoral nature of the interventions conducted with different aims, navigating these aims through barriers within country laws, practices and standards, restricted synthesis, different population studies (camp based and urban-based refugees) and study designs lacked comparative data for the most part. Overall, there was a general oversight on the discussion about laws in Jordan impacting the implementation of the MISP for Syrian refugees. Inclusion of grey literature resulted in inclusion of many self-reported data by agencies, which might imply high risk of bias. Limited data was available on the current state of MISP implementation in Jordan almost 9 years post crisis.

It should also be noted that many of the aforementioned limitations are to be expected, given the nature of humanitarian-context, where often conducting a thorough research is crippled given the many context challenges as well as the inherent vulnerabilities of displaced women and girls as a result of fear, stigma and the need for secrecy [37]. The authors, however, believe this review helps in providing a comprehensive overview of the existent barriers to SRH use and service delivery in Jordan 9 years into the crisis. It also guides in providing evidence-based recommendations.

## Conclusions

The MISP is a set of priority activities and services that are recognized as standard care for SRH in emergency and humanitarian settings. Since the beginning of the Syrian crisis, the influx of the Syrian refugee population into Jordan increased rapidly over the years. Hence, information and data on the needs and challenges of this population is rapidly becoming outdated and requires regular monitoring. Any SRH program must integrate the needs of Syrian refugees residing within host communities in addition to those living in camps. Despite the significant progress in the MISP policies and the review of the IAFM guidelines in 2018 at a global level, there remains gaps in the overall availability and utilization of the different MISP and SRH service delivery in Jordan.

Furthermore, this review spotted the light on the limited research conducted around MISP implementation and its delivery in Jordan. In fact, only one study by Doedens et al., that was conducted in 2013 [33] and then published in 2015 [4], evaluated the implementation of the MISP objectives, effectiveness of the coordination mechanisms, and training on the mentioned package in Jordan. Most of the available literature discussing MISP cited one or both of these two sources. Hence, to be able to inform national SRH guidelines and policies in Jordan for improved SRH service delivery to respond to the needs of this refugee population without compromising the needs of the general Jordanian population, more implementation research is needed to identify barriers and challenges as well as inform evidence-based strategies to improve Syrian refugees’ access to SRH services.

## Data Availability

All data generated or analyzed during this study are included in this published article and/or its supplementary information files.

## Abbreviations

AIDS: Acquired Immune Deficiency Syndrome
ANC: Ante-Natal Care
ART: Anti-Retroviral Therapy
ARV: Anti-Retroviral
BEmONC: Basic obstetric and newborn complications
BMC: Bio-Med Central
BMJ: British Medical Journal
CEmONC: Comprehensive Emergency Obstetric and Newborn Care
CMR: Clinical Management of Rape
EC: Emergency Contraception
EMHJ: Eastern Mediterranean Health Journal
EmONC: Emergency obstetric and Newborn Care
FGD: Focus Group Discussion
FP: Family Planning
FV: Family Violence
GBV: Gender-Based Violence
HIV: Human Immunodeficiency Virus
IEC: Information Education and Communication
IPV: Intimate Partner Violence
IUD: Intra-Uterine Device
JCAP: Jordan Communication Advocacy and Policy activity
KI: Key Informant
MISP: Minimum Initial Service Package
MoH: Ministry of Health
NGO: Non-Governmental Organizations
PEP: Post-exposure prophylaxis
PRISMA: Preferred Reporting Items for Systematic Reviews and Meta-Analyses
PMTCT: Prevention of mother-to-child transmission
RH: Reproductive Health
SDGs: Sustainable Development Goals
SGBV: Sexual and Gender Based Violence
SRH: Sexual and Reproductive Health
STI: Sexual Transmitted Infections
SV: Sexual Violence
UN: United Nations
UNFPA: United Nations Population Fund
UNHCR: United Nations High Commissioner for Refugees
USAID: United States Agency for International Development
